# Anthocyanins and Carotenoids Characterization in Flowers and Leaves of Cyclamen Genotypes Linked with Bioactivities Using Multivariate Analysis Techniques

**DOI:** 10.3390/antiox11061126

**Published:** 2022-06-07

**Authors:** Mihaiela Cornea-Cipcigan, Andrea Bunea, Cosmina Maria Bouari, Doru Pamfil, Emőke Páll, Adriana Cristina Urcan, Rodica Mărgăoan

**Affiliations:** 1 Department of Horticulture and Landscaping, Faculty of Horticulture, University of Agricultural Sciences and Veterinary Medicine, 400372 Cluj-Napoca, Romania; mihaiela.cornea@usamvcluj.ro; 2 Department of Chemistry and Biochemistry, University of Agricultural Sciences and Veterinary Medicine, 400372 Cluj-Napoca, Romania; andrea.bunea@usamvcluj.ro; 3 Department of Microbiology, Immunology and Epidemiology, Faculty of Veterinary Medicine, University of Agricultural Sciences and Veterinary Medicine, 400372 Cluj-Napoca, Romania; cosmina.bouari@usamvcluj.ro; 4 Research Centre for Biotechnology in Agriculture Affiliated to Romanian Academy, University of Agricultural Sciences and Veterinary Medicine, 400372 Cluj-Napoca, Romania; presedinte.arfc@academia-cj.ro; 5 Department of Clinical Sciences, University of Agricultural Sciences and Veterinary Medicine, 400374 Cluj-Napoca, Romania; emoke.pall@usamvcluj.ro; 6 Department of Microbiology and Immunology, Faculty of Animal Science and Biotechnologies, University of Agricultural Sciences and Veterinary Medicine, 400372 Cluj-Napoca, Romania; adriana.urcan@usamvcluj.ro; 7 Laboratory of Cell Analysis and Spectrometry, Advanced Horticultural Research Institute of Transylvania, University of Agricultural Sciences and Veterinary Medicine, 400372 Cluj-Napoca, Romania

**Keywords:** cyclamen, bioactive compounds, antioxidant activities, antimicrobial activities, cytotoxicity assay, multivariate statistics

## Abstract

The present study was carried out to evaluate and compare in vitro antioxidant (2,2-diphenyl-1-picrylhydrazyl (DPPH), Trolox equivalent antioxidant capacity (TEAC), and ferric reducing antioxidant power (FRAP)), antimicrobial, anticancer activities, and the individual carotenoids and anthocyanins content of methanol extracts of the Cyclamen genotypes: Persian cyclamen accessions (*Cyclamen persicum* Mill.), sowbread (*C. mirabile* Hildebr.), and ivy-leaved cyclamen (*C. hederifolium* Mill.) aerial parts. The HPLC-PDA analysis revealed the presence of five individual carotenoids (i.e., neoxanthin, violaxanthin, lutein, β-carotene, and cis-β-carotene) as the main compounds in *Cyclamen* leaves, and the presence of seven individual anthocycanins (i.e., cyanidin 3,5-di-*O*-glucoside, peonidin-rutinoside, peonidin 3,5-di-*O*-glucoside, peonidin 3-*O*-glucoside, malvidin 3-*O*-glucoside, malvidin 3,5-di-*O*-glucoside, and malvidin-rutinoside) in Cyclamen flowers reported, hereby, for the first time. The highest phenolic content was found in the leaves of LC6, *C. mirabile* (46.32 ± 0.14 mg/g gallic acid equivalents [GAE]), and in the flowers of *C. persicum* Merengue Magenta (FC15) (58.63 ± 0.17 mg/g GAE), whereas the highest flavonoid content was reported in *C. persicum* Halios Falbala leaves, namely LC9 (54.90 ± 0.27 mg/g quercetin equivalents [QE]) and in flowers of *C. persicum* Victora (FC2) (77.87 ± 0.25 mg/g QE). The highest antioxidant activity in DPPH and FRAP assays was reported in *C. persicum* Dark Violet (LC1) and Victoria (LC2), whereas *C. mirabile* (LC6) had the highest activity in the TEAC assay. In flowers, high antioxidant activities in DPPH and TEAC were noticed in *C. persicum* Superserie Red (FC7) and Dark Violet (FC1), respectively, and Halios Falbala (FC9) exhibited the highest activity in the TEAC assay. Additionally, FC9 exhibited the highest antibacterial activity in almost all tested bacteria compared with the leaves extracts. Furthermore, the highest in vitro citotoxicity in MDA-MB-231 cells was noticed in *C. hederifolium* LC18 (56.71–69.35%) and FC18 (40.07–41.43%), with a lower effect against BJ cells demonstrating selective toxicity. The above findings, highlight the potential use of the Cyclamen flower and leaf extracts as significant anticancer agents along with their antioxidant and antimicrobial properties.

## 1. Introduction

As estimated by The World Health Organization [1], 60% of herbal drugs may increase in the next 20 years, as well as 80% of people in developing countries (65% of the world’s population) that are still relying on traditional medicine. The Cyclamen genus comprises 24 species and is cultivated in the Mediterranean regions with temperate climates [2], from which the purple cyclamen (*C. purpurascens* Mill.) is decreasing due to its collection for medicinal purposes in Croatia [3,4], and Lebanon cyclamen (*C. libanoticum* Hildebr.) is already endangered in accordance to the International Union for the Conservation of Nature (IUCN) Red List [5]. The medicinal properties of Cyclamen have been known since antiquity by Romans, Greeks, and Egyptians. As a traditional medicine, the crushed tubers are used to cover infected wounds, psoriases, eczema, ulcers, and other skin disorders [6,7]. It is used as an anti-helminthic, laxative, anti-arthritic, and anti-rheumatic [8], whereas dried leaves are used to cure minor skin burns and disorders [9]. Traditionally, tubers of *C. persicum* were used as a treatment against arthritic and rheumatic disorders, whereas the Eastern sowbread (*C. elegans* Boiss. and Buhse.) was used against headaches, muscle contractions, and toothaches. Nowadays, due to their bioactive compounds, they are extensively used as ornamental and therapeutic plants [10,11,12]. *C. hederifolium* has been used in several regions of Serbia as a treatment against rheumatism, skin conditions, menstrual pains, and migraines, but also as a purgative and antitumor agent. Traditional Italian medicine utilizes tubers as a treatment for frostbites, warts, and hemorrhoids, whereas in Turkey, several species are used against infertility [2,13,14]. *C. mirabile* is used as an antifungal agent against *Candida* sp. and *Cryptococcus neoformans* [13].

Over the past decades, the diet–cancer association has been significantly studied. In particular, epidemiological reports linked diet with cancer frequency and assertiveness. Among them, some carotenoids have been demonstrated to possess protective effects against the occurrence of a large variety of cancer types. Even though these correlations might not involve fundamental relationships, patterns connected with a lower incidence of cancer can be identified. Carotenoids are characterized as a diverse and abundant assembly of natural pigments present in high amounts in plants, vegetables, and fruits [14,15,16,17]. They are responsible for plant photoprotection (adaptation to light stress) and protect the cells against damage induced by light and superoxide radicals [18]. Among biological characteristics, β-carotene and β-cryptoxanthin are recognized for their pro-vitamin A activity, a key role in human health [19], whereas xanthophylls (lutein, neoxanthin, and violaxanthin) have photoprotective functions [20]. The antioxidant capacity of carotenoids are recognized for their health-promoting characteristics in plants and fruits [21]. The intake of carotenoids has been associated with lower risks of degenerative diseases, counting gastrointestinal tract, lung, skin, breast, and prostate cancers [22,23,24,25,26,27,28]. Anthocyanins are water-soluble pigments, having a broad assortment of colors, such as orange, pink, red, blue, and purple hues, based on the environmental pH [29]; their biosynthesis is strongly affected by stress conditions (i.e., photoprotection) and they directly scavenge reactive oxygen species (ROS) to reduce the leaves’ oxidative damage [30]. Recent studies stated the relationship between the dietary anthocyanins intake, protection against neurological diseases, reversed age-related brain and cognitive functions, reduced risk of heart disease, and anti-inflammatory roles which can also act as anticancer agents [31]. Flower color is a distinct characteristic of Cyclamen morphology. In the present study, the genotypes possessing silvery foliage, a fringed type, and novel flower pigments were chosen as based on the fact that these characteristics are gaining interest in the floricultural field. From the literature search, there were no reported data regarding the individual carotenoids in *Cyclamen* species, whereas detailed analyses regarding the anthocyanins in flowers were only reported in *C. purpurascens* [32,33,34]. Furthermore, several research projects attempted to create novel pigments in Cyclamen by the use of specific enzymes for altering the existing pigments or by ion-beam irradiation and evaluating their expression patterns by PCR analysis [35,36,37]. Few molecular studies have been reported regarding the anthocyanin biosynthesis and flower pigments in Cyclamen [37]. *Cyclamen* species might be potential agents against the treatment of several types of cancer, due to their inhibition and/or induction of a co-administered drug metabolism, but also in the induction or suppression of the CYP450s-dependent drug metabolizing enzymes. In this aspect, Arslan and Ozgun demonstrated that water extracts of *C. trochopteranthum* tubers demonstrated moderate cytotoxic activity in HepG2 and Caco-2 cell lines. In addition, the induction of CYP450s’ mRNA levels in HepG2 and the suppression in the Caco-2 cell lines were noticed, demonstrating tissue-specific responses [38]. In a different study, triterpene saponins isolated from the tubers of *C. hederifolium* were subjected to several cancel cell lines with no significant reduction in cell number [39]. The same was noticed using isolated triterpene saponins from *C. persicum* which demonstrated a low cytotoxicity against two human colorectal cancer cell lines, namely HCT 116 and HT–29. Thus, the anticancer properties of Cyclamen prove to be species-specific, but also based on plant material and extraction procedure [40,41,42,43]. As there are no comprehensive studies examining the individual carotenoid and anthocyanin profiles of these genotypes, the current study explores the possible correlation between carotenoids, anthocyanins, and flower color space values counting L* (lightness), a*, b*, C* (chrome), and *H*° (shade), which will be a starting point to establish a fast, cost-effective, and accurate pigment content estimation. Furthermore, by the use of multivariate analysis, the correlation between isolated compounds and bioactivities (i.e., antioxidant, antimicrobial activities, and cytotoxicity assays) was evaluated. Therefore, the main purposes of this study were to (i) determine the total phenolic, flavonoid, anthocyanins, and carotenoids content from the leaves and flowers; (ii) identify and quantify the individual carotenoids and chlorophylls in the leaves, and the individual anthocyanins in the flowers of Cyclamen; (iii) determine the antioxidant, antimicrobial, and antitumoral activities; and (iv) determine the correlation between the above-mentioned activities and identified compounds using multivariate analysis.

## 2. Materials and Methods

### 2.1. Chemicals and Reagents

HPLC grade acetonitrile, formic acid, ethyl acetate, petroleum ether, and diethyl ether were purchased from Sigma-Aldrich (Steinheim, Germany). Chlorophyll a and b standards were purchased from Sigma-Aldrich (Steinheim, Germany). Lutein, β-carotene, violaxanthin, and neoxanthin standards were purchased from Merck, Germany. Trolox, DPPH, and trypsin-EDTA were purchased from Sigma-Aldrich (Steinheim, Germany). Mueller–Hinton (MH) agar was purchased from Merck (Germany). DMSO (dimethyl sulfoxide) was purchased from Fluka (Buchs, Switzerland).

### 2.2. Plant Material and Sample Preparation

**Figure 1:**
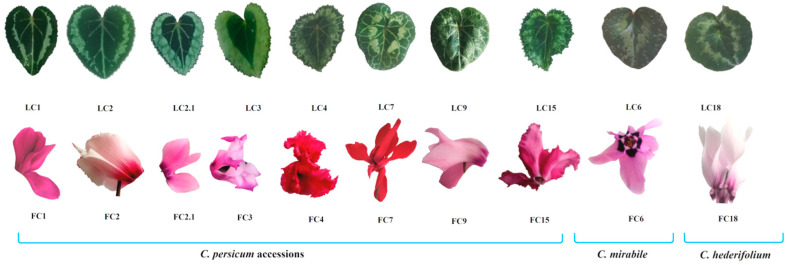
Cyclamen genotypes used in the study (LC—*Cyclamen* leaves; FC—Cyclamen flowers). *C. persicum* accessions: C1—Superserie Dark Violet; C2—Victoria; C2.1—Violet fonce; C3—Origami; C4—Merengue Salmon red; C7—Superserie Red; C9—Halios Falbala; C15—Merengue Magenta; C6—*C. mirabile*; C18—*C. hederifolium*.

Seeds of *C. persicum* were granted by the Morel Diffusion SAS (Fréjus, France), whereas *C. mirabile* seeds were acquired from the Mendel University Botanical Gardens and Arboretum (Brno, Czech Republic), and *C. hederifolium* seeds from University of Jena Botanical Gardens (Jena, Germany) (Figure 1).

After reaching maturity, flowers and leaves samples were collected and the extract preparation was made from dry samples after lyophilization. The freeze-drying process was carried out to determine the water content and compare the experimental results with literature data often expressed as dry weight [44,45,46]. The dry samples were ground and homogenized, after which a quantity of 1 g of each sample was weighed. Samples were extracted individually with 10 mL of solvent (methanol: water, 80:20), according to previous reports regarding best extraction methods [47,48]. The mixture was kept in a Bandelin ultrasonic bath for 2 h. The resulting extract was centrifuged at 3500× *g* for 20 min. The supernatant was removed, collected in Eppendorf tubes and stored in the freezer at −18 °C until analysis was performed. All assays were measured in triplicate and the results are given as mean ± standard deviation. The extracts were further assessed for determining the antioxidant, antimicrobial, and cytotoxic assays. For the determination of chloropylls, carotenoids, and anthocyanins, different extracts were utilized (see Section 2.4, Section 2.5 and Section 2.6).

### 2.3. Color Measurements

The color of *Cyclamen* leaves and flowers was measured using Konica Minolta (Sensing Americas) CM-700d portable spectrophotometer. The L* coordinate expresses the sample’s lightness (white = 100 and black = 0), a* coordinate position on the red–green axis (red = positive, green = negative) and b* coordinate on the yellow–blue axis (yellow = positive, blue = negative). From these values the color intensity (chroma, *C*) and shade (hue angle, *H°*) [49] were calculated using the following equations:

*C* = (a*^2^ + b*^2^)^0.5^ (1)

*H*° = tan^−1^ (b*/a*) (2)

### 2.4. Chlorophyll Content

Chlorophyll content was evaluated using a previously published method [50]. Fresh plant samples (0.2 g) were grinded and extracted with acetone (90%). After filtration, the extraction process was made until the residue became colorless. For determining the chlorophyll a (Chl a) and chlorophyll b (Chl b) content, the absorbance was measured at 645 and 663 nm using the V-530 UV-VIS Spectrophotometer (Jasco, Eason, MD, USA) and the concentrations were quantified using the formulas:

Chl a (mg/g) = (11.75 × A^663^–2.35 × A^645^) × V/g  Chl b (mg/g) = (18.61 × A^645^–3.96 × A^663^) × V/g (3)

where A645 and A663 correspond to the optical density at a particular wavelength, V corresponds to the extract’s volume (mL), and g corresponds to the sample’s weight (mg).

### 2.5. HPLC-PDA Analysis of Carotenoids

To the best of our knowledge there are no reports regarding the determination of individual carotenoids in Cyclamen, therefore, this extraction step was carefully performed to reduce loss of pigments. The carotenoids extraction starts from 2 g of fresh leaf samples, made with methanol/petroleum ether/ethyl acetate (1:1:1) mixture, and performed until the solvents became colorless. The combined extracts were separated with saturated saline solution and diethyl ether. The upper organic phase was evaporated and the obtained residue was dissolved in known volumes of diethyl ether and potassium hydroxide (30%) in methanol for saponification. After separation, the organic phase was dried until evaporation. For the individual carotenoids quantification, ethyl acetate was used to dilute the samples, followed by filtration and analyzation using a Shimadzu HPLC system equipped with degasser DGU-20 A3 and LC-20 AT binary pump (Prominence), SPD-M20 photodiode array detector (HPLC-PDA), and YMC C30 (24 cm × 4.6 mm, 5 µm particle size) column for the separation of carotenoids. The mobile phase was at 0.8 mL/min flow rate. Solvent A: methanol/tert-butyl methyl ether/deionized water (81:15:4, *v/v/v*). Solvent B: tert-butyl methyl ether/methanol/deionized water (90:7:3, *v/v/v*) [51]. Carotenoids identification was achieved by comparison of UV–VIS spectra and retention time of sample peaks with those of standards (lutein, β-carotene, violaxanthin, and neoxanthin).

### 2.6. HPLC-PDA Analysis of Anthocyanins

The determination of antocyanins from the genotypes used in the present study are reported hereby for the first time, thus the extraction step was carried out as to decrease the loss of pigments. The extraction of anthocyanin compounds was performed from 1 g of cyclamen flower samples in acidified methanol (HCl 0.3%, (*v/v*)) using a homogenizer (Miccra D-9 KT Digitronic, Bergheim, Germany). The re-extraction was performed until the color of the extraction solvent disappeared, the final extraction being performed in darkness at 4 °C. Separation was carried out on the same HPLC system equipped with a UV-VIS detector and a Luna Phenomenex C-18 column. The used mobile phase was formic acid (4.5%) in double distilled water (solvent A) and acetonitrile (solvent B). The flow rate was 0.8 mL/min, and analyses were carried out at 35 °C. Data were measured at 520 nm. The quantification of anthocyanins was performed using malvidin-3-glucoside as standard, in the concentration range of 2.5–500 µg/mL, and the linearity of the calibration curve was *R*^2^ > 0.998.

#### HPLC-DAD-ESI^+^ for Anthocyanins Identification

The Agilent 1200 HPLC system equipped with quaternary pump, auto-sampler, solvent degasser, and UV-Vis detector with photodiode (DAD) attached to single-quadrupole mass detector 6110 (MS) (Agilent Techologies, CA, USA) was used. Separation of compounds was carried out using the Eclipse XDB C18 column (Agilent Techologies, CA, USA), using mobile phases A (Water + Acetic Acid 0.1%) and B (Acetonitrile + Acetic Acid 0.1%) in the gradient below, at 25 °C for 30 min, and a 0.5 mL/min flow rate. Chromatograms were recorded at wavelengths *λ* = 280, 340 and 520 nm. For MS, the ESI^+^ ionization mode was used accordingly: capillary voltage 3000 V, at 350 °C, nitrogen flow 7 L/min, nebulization pressure 35 psi, fragmentary voltage 100 eV, and *m/z* 120–1200, full-scan. Acquisition and interpretation of data was done using Agilent ChemStation software (version B.04.02, Agilent Technologies, Hewlett-Packard-Strasse 8 76,337 Waldbronn).

### 2.7. Determination of Total Phenolic Content (TPC) and Total Flavonoid Content (TFC)

The TPC was evaluated using Folin–Ciocalteu method [52]. In a volume of 10 µL of the studied samples, 100 µL Folin–Ciocalteu reagent (0.2 N) and 80 µL sodium carbonate (Na_2_CO_3_) solution (1 M) were added. The absorbance was read at 765 nm. Gallic acid was used as standard for the quantification of TPC and the calibration curve was between 0.025–0.15 mg/mL (*R*^2^ = 0.9992). There was TPC distilled water (100 µL) and 10 µL of sodium nitrate (5% NaNO_2_) solution. After 5 min, they were expressed as mg of GAE per g of dw (dry weight) sample. Flavonoids were measured using the aluminum chloride colorimetric method with slight modifications [53,54]. From each sample, 25 µL extract was added to 15 µL of aluminum chloride (10% AlCl_3_). Finally, the same volume (50 µL each) of sodium hydroxide (1 M) and distilled water were added. The absorbance was read at 510 nm. The calibration curve of quercetin was between 0.025–0.2 mg/mL (*R*^2^ = 0.9987). The TFC was expressed as mg of QE per g of dw sample.

### 2.8. Antioxidant Activity

#### 2.8.1. Free-Radical-Scavenging Assay (DPPH)

The scavenging activity of cyclamen leaf and flower extracts against DPPH was spectrophotometrically evaluated after [16] with slight modifications. Briefly, an aliquot (40 µL) of each extract was added to 200 µL DPPH solution and incubated for 15 min in the dark at room temperature. Absorbance was read at 517 nm. The radical scavenging activity was expressed as mg equivalent Trolox per g of dw sample.

#### 2.8.2. ABTS Radical Scavenging Activity

The ABTS radical scavenging activity assay was carried out according to [55] with some modifications. The ABTS*^+^ was produced by the reaction between ABTS solution (7 mM) and potassium persulfate solution (2.45 mM), incubated overnight at room temperature for 12 h. The ABTS*^+^ solution was mixed with 17 µL of extract.

For ABTS radical scavenging activity the percentage of absorbance inhibition at 734 nm was calculated using the equation below:

Inhibition (%) = [1 − A_sample_/A_blank_] × 100 (4)

Percentages of inhibition of the samples were then compared with a standard curve made from the corresponding readings of Trolox (0.4–0.04 mg).

Results were expressed in mg of equivalent Trolox per g of dw sample.

#### 2.8.3. Determination of Ferric Reducing/Antioxidant Power (FRAP)

The evaluation of FRAP was carried out using the method described by [56] with the adaptations imposed by the matrix studied. For the determinations, 300 µL FRAP reagent, 10 µL methanolic extract of cyclamen leaf and flower, and 10 µL of ultrapure water were used. The samples were incubated at 37 °C for 5 min. The antioxidant capacity was analyzed corresponding to the reaction signal provided by known concentration of aqueous Fe^II^ solutions (0.1–1 mmol/L of FeSO_4_·7H_2_O) and using a standard calibration curve (*R*^2^ = 0.9965). The absorbance was read at 593 nm and results expressed as mmol/g Fe^II^ dw sample.

### 2.9. Antimicrobial Activity—In Vitro Qualitative Study

The antimicrobial activity of the cyclamen extracts was in vitro evaluated by the disk diffusion test, according to the EUCAST standard method [57,58], designed to identify the extracts with increased antimicrobial potential. For this study, standard strains of Gram-positive and Gram-negative bacteria, and yeasts were selected. Gram-positive bacteria were represented by: *Bacillus cereus* ATCC 11778, *Enterococcus fecalis* ATCC 29212, *Listeria monocytogenes* ATCC 13932, and *Staphylococcus aureus* ATCC 6538P. Gram-negative bacteria used were represented by: *Escherichia coli* ATCC 10536, *Klebsiella pneumoniae* 14930, and *Salmonella enteritidis* ATCC 13076, while the yeast strain was *Candida albicans* ATCC 10231. Amoxicillin, Norfloxacin, and Miconazole were used as standard antibacterial and antifungal controls. The bacterial strains were inoculated separately on MH agar plate and incubated for 24 h at 37 ± 2 °C, while Sabouraud dextrose agar (SDA) was used for Candida yeast. Afterward a standardized inoculum of the tested microorganism in a sterile saline solution, with an adjusted 0.5 optical density on McFarland scale using Densichek device (bioMérieux, France) was prepared. Plastic Petri dishes containing MH agar for bacteria and SDA agar for yeast (8.5 cm diameter) were inoculated with 500 µL of the microbial suspension over the entire agar surface. The excess fluid was removed and the agar surface allowed to dry at 35 °C for 15–20 min. Afterward, seven radial holes of 5 mm diameter were punched aseptically and 20 µL of the sample extract (FC6, FC9, FC18, LC6, LC9, and LC18) was distributed into each well. As a negative control, the solvent for bioactive compounds extraction (methanol 80%) was used, and as positive control, Amoxicillin (30 μg/mL) for Gram-positive, Norfloxacin (10 μg/mL) for Gram-negative, and Miconazole (10 μg/mL) for yeast were used. The plates were incubated at 35 ± 2 °C for 18 h for bacteria and at 28 °C for 48 h for the fungal strain, and the diameter of the inhibition zones (in mm) was measured.

### 2.10. Cytotoxicity Assay

The cytotoxicity assay of cyclamen methanolic extracts were performed using human fibroblasts BJ and human breast adenocarcinoma MDA-MB-231 cell lines. The standard conditions were used for cultured cells. The (4,5-dimethylthiazol-2-yl)-2,5-diphenyltetrazolium bromide (MTT) assay [59] was assessed for determining the potential cytotoxicity of cyclamen extracts. To obtain cell suspensions, trypsin-EDTA (0.25%) was used to treat the cells. After centrifugation for 5 min at 1500× *g*, after which 1 × 10^4^ cells/well were seeded in 200 µL complete culture medium on 96-well plates. After 24 h, six Cyclamen extracts in three different concentrations were added. Doses for testing were determined based on the concentration of TPC mg/g GAE in the studied samples (FC6, FC9, FC18, LC6, LC9, and LC18). Untreated cells represent the control samples. The medium was removed after 24 h and 100 µL of 1 mg/mL MTT solution was added. After incubation at 37 °C in darkness for 3 h, the MTT solution was removed and 150 µL of DMSO solution was added. Cell proliferation analysis was carried out after 24 h by spectrophotometric readings at 450 nm using the BioTek Synergy 2 microplate reader (Winooski, VT, USA). Data are shown as optical density or average proliferation percentage rate compared with the control of untreated cells.

### 2.11. Multivariate Statistics

Statistical significance was evaluated by one-way analysis of variance (ANOVA) and post hoc Tukey test with XLSTAT software (Addinsoft, New York, NY, USA). Hierarchical cluster analysis (HCA) and principal component analysis (PCA) were performed using the Paleontological Statistics (PAST) software (version 4.0, Oslo, Norway) [60], and Pearson’s correlation was carried out using XLSTAT software (Addinsoft, New York, NY, USA). HCA was performed on a Bray–Curtis similarity with complete linkage. Heatmap and dendrograms were generated using the Euclidean distance based on Ward’s algorithm for clustering [61].

## 3. Results and Discussion

### 3.1. Colorimetric Evaluation and Classification

The color of flowers and leaves is a phenotypic characteristic which usually indicates the plant’s physiological status. The color of leaves indicates the accumulation of secondary metabolites with significant chromatic constituents, which might possess ornamental, therapeutic, and commercial significance. Furthermore, the variation of the pigment provides data regarding the genetic intra- and inter-specific variability of populations and/or taxa, acknowledging the development of evolutionary theories from a phylogenetic point of view. To precisely evaluate the color of *Cyclamen* leaves and flowers, the color parameters were measured, which may help in discriminating similar genotypes based on their color measurements.

Data obtained by the colorimetric analysis of Cyclamen genotypes were reported in Table 1. In leaves, the luminance L* ranged between 37.83 (LC7) and 76.67 in *C. hederifolium* (LC18), the redness a* between −5.4 in *C. mirabile* (LC6) and −27.7 (LC2.1), and the yellowness b* between 40.90 (LC9) and 11.30 (LC7), with significant differences. In flowers, the luminance L* ranged between 56.7 (FC9) and 26.8 (FC1), the redness a* between 55.6 (FC2.1) and 31.2 (FC2), and the yellowness b* between 27.15 (FC7) and −16.85 (FC3), with significant differences. Higher values in L* indicate increased luminosity of *C. hederifolium* leaves and flowers being a lighter variety with lower values in a*. In contrast, the dark colored genotypes (FC1, FC4, and FC7, respectively) exhibited lower parameters for the L* values in flowers and leaves, but also higher values in the other flowers and lower in leaves. Meanwhile, in darker colored flowers the values of b* significantly decreased and increased in leaves. Overall, the palest genotype with the highest L* in both leaves and flowers proved to be *C. hederifolium* (C18), whereas the darker colored was *C. persium* Superserie Red (C7). The highest and lowest values in redness a* were noticed in the flowers and leaves of Violet fonce (C2.1). The highest values in color intensity were shown in the leaves of Halios falbala (C9) and flowers of Violet fonce (C2.1) and the lowest in *C. hederifolium*, as noticed by the highest values in L*. Similar results were reported in *C. purpurascens* with higher values of L* in semi-silvery and patterned leaves, compared with green and silvery leaf lamina. In accordance with the study’s results, all genotypes exhibited positive b* values, suggesting a slightly yellow hue in the leaves [32]. Furthermore, increased *H*° values were also reported in the flowers of *C. purpurascens* and lower values in the leaves [34]. Consistent with previous reports, higher values in a* were observed in *C. mirabile* leaves (LC6), denoting a tendency of purple coloration in the marbled and semi-silvery genotypes, whereas in *C. persicum* Violet fonce (FC2.1), the flowers indicated an accumulation of red and purple pigments. In contrast, lower levels in a* denote a tendency of pink and red coloration.

### 3.2. Chlorophyll Content

Chlorophylls are natural pigments with a significant function in plant growth, as a vitamin supply, for disease resistance, detoxification, and having several health benefits, such as lower risks of cancer development, being anti-inflammatory, and antioxidant activities [62].

**Figure 2:**
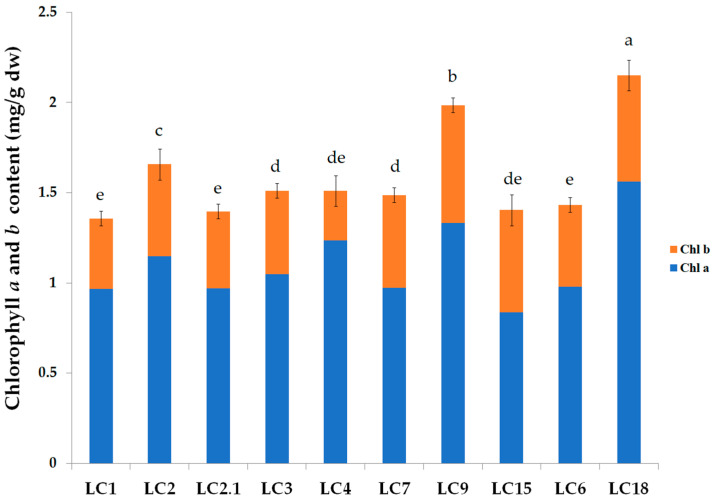
Chlorophyll a and b content in *Cyclamen* leaves. Different letters indicate significant differences (*p* < 0.05).

Significant differences in the content of chlorophyll a and b were observed in all *Cyclamen* genotypes (*p* < 0.05). The highest concentrations of chlorophyll a were found in *C. hederifolium* (LC18), 1.563 ± 0.2 and 1.333 ± 0.21 mg/g dw (LC9), whereas high concentrations of chlorophyll b were found in LC9 (0.654 ± 0.12 mg/g dw) and LC18 (0.587 ± 1.09 mg/g dw) (Figure 2). The lowest concentrations of chlorophyll a were noticed in LC15 (0.613 ± 0.09 mg/g dw) and the lowest chlorophyll b in LC4 (0.206 ± 0.19 mg/g dw). As previously reported, the higher content of chlorophyll in the *Cyclamen* species might be related to the darker green leaf sections and the lower silver-leaf sections [32,63]. In a different study, variations in the chlorophyll content proved to be significantly different based on the collection site and period of collection. Thus, a higher chlorophyll content was noticed in the April-collected *C. purpurascens* leaves compared to the ones collected in February which showed significantly lower levels. Changes in the pigment concentrations might be due to variations in light intensity according to each season [63]. In a different study, the silver *C. purpurascens* genotypes contained significantly lower levels of chlorophyll compared with the green or marbled leaves type [32]. Changes in the chlorophyll content significantly differ among genotypes from a physiological and molecular point of view, but also based on geographical regions, and climatic and soil conditions.

### 3.3. Qualitative and Quantitative Analysis of Individual Carotenoids in Cyclamen Leaves

In multiple flowers and fruits, carotenoids are accountable for the yellow, orange, and red pigments. In green leaves and leafy vegetables which contain higher amounts of chlorophyll, the color of carotenoids is masked despite its significant content [64]. Due to their antioxidant properties, carotenoids have been associated with the prevention of several types of cancer development [65], cardiovascular and neurodegenerative diseases [66], chronic psychological stress [67], inflammation, as well as macular degeneration [68,69,70,71].

**Figure 3:**
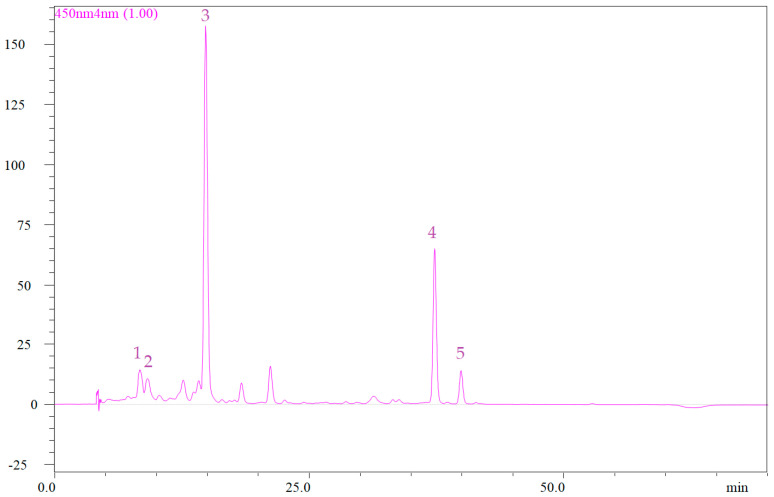
HPLC chromatogram of carotenoids identified from *C. persicum* leaves (LC1). Peak no. 1, neoxanthin; 2, violaxanthin; 3, lutein; 4, β-carotene; 5, cis-β-carotene.

The quantification of individual carotenoids is hereby, firstly reported in the scientific literature for the *Cyclamen* species used in this study, representing a basis for future studies to demonstrate the importance of their biological activities and therapeutic properties. A total of five carotenoids were detected in the leaves of *Cyclamen* cultivars, as well as in *C. hederifolium* and *C. mirabile*, identified as: 1 neoxanthin, 2 violaxanthin, 3 lutein, 4 β-carotene, and 5 cis-β-carotene (Table 2, Supplementary Figure S1). Neoxanthin is a carotenoid responsible for the protection against photo-oxidative stress [72]. The content of neoxanthin (peak 1) ranged from 6.05 ± 0.31 μg/g fw (LC2.1), 7.27 ± 2.7 dw (LC3) in *C. persicum* accessions, and 21.49 ± 3.14 μg/g fw and 31.13 ± 2.2 dw (LC6) in *C. mirabile* (Figure 3).

Violaxanthin is biosynthesized from zeaxanthin and is involved in the elimination of excessive light energy. It also demonstrated increased lipid peroxidation-inhibitory activity compared with β-carotene [73].

Violaxanthin (peak 2) was identified in the highest amount in *C. mirabile* (15.52 ± 1.29 μg/g fw and 22.48 ± 0.87 μg/g dw), followed by *Cyclamen* accessions LC4 (14.49 ± 0.23 μg/g fw) and LC9 (20.01 ± 2.87 μg/g dw). The lowest amounts were noticed in *C. hederifolium* (7.74 ± 0.61 μg/g fw and 11.96 ± 0.35 μg/g dw) and in the other *Cyclamen* accessions, particularly LC3 (5.53 ± 0.23 μg/g dw).

Lutein (peak 3) was identified as the major compound in the studied species and accessions, but the content was significantly different (*p* < 0.05) between species. The lowest amount of lutein (31.91 ± 1.6 μg/g fw and 44.92 ± 0.45 μg/g dw) was noticed in LC3, followed by a closer value (36.92 ± 1.5 μg/g fw and 49.15 ± 0.43 μg/g dw) in LC4. The highest quantity (90.68 ± 9.05 μg/g fw) was observed in LC9, followed by *C. hederifolium* (87.42 ± 9.23 μg/g fw). Similar to *Cyclamen* species, in *Calendula officinalis* L., the main identified carotenoids were lutein (34.93%), β-carotene (26.70%), and violaxanthin (10.02%) [74]. On the contrary, in *Cynodon dactylon* (L.), an Indian ornamental and medicinal plant, the predominant carotenoid was β-carotene (35.2 ± 0.72 mg/100 g), followed by lutein (17.0 ± 0.33 mg/100 g) and lower amounts of violaxanthin and zeaxanthin [75]. Additionally, in *Adiantum capillus-veneris* leaves, 9′-Z-neoxanthin (142.8 μg/g) and all-E-violaxanthin (82.2 μg/g) were present in the highest amount [76]. Several reports demonstrated that lutein supplementation lowered the risk of development to late age-related macular degeneration in patients between 50 and 85 years by about 25% [77], whereas supplementation with 10–20 mg/day of lutein was associated with increased macular pigment optical density, contrast sensitivity, and visual acuity [78]. Furthermore, lutein intake influenced the anthropometric indices, metabolic parameters, and body composition in overweight middle-aged patients. The total cholesterol of serum levels and visceral fat, and LDL-cholesterol considerably decreased in individuals who consumed 20 mg/day of lutein for 2 weeks [79].

β-carotene (peak 4), is a fat-soluble compound and natural pigment found in nature, as well as an important source of vitamin A [80]. Abundant in Cyclamen genotypes, it is an antioxidant with detoxifying effects and an essential nutrient for maintaining human health by lowering the incidence of atherosclerotic cardiovascular disease [81,82]. The highest content was found in LC4 (97.09 ± 0.23 μg/g fw and 129.26 ± 12.6 μg/g dw), followed by *C. hederifolium* (35.85 ± 5.45 μg/g fw and 55.40 ± 2.8 μg/g dw), whereas the lowest values were found in LC3 (12.67 ± 5.12 μg/g fw and 17.83 ± 4.6 μg/g dw), followed by LC15 (13.09 ± 0.97 μg/g fw and 17.85 ± 3.12 μg/g dw), respectively. Lastly, cis-β-carotene was found in higher amounts in *C. hederifolium* (10.46 ± 0.88 μg/g dw) and in LC4 (7.47 ± 0.09 μg/g fw), whereas the lowest values were found in LC15 (3.55 ± 1.1 μg/g dw and 2.61 ± 0.21 μg/g fw). No data were found in the literature on the composition of carotenoids for the *Cyclamen* species. Consequently, the contribution of this study is relevant to better understand the composition of these genotypes of significant importance for the ornamental sector and for medicinal purposes. In the present study, the highest carotenoid content was noticed in *C. persicum* Merengue Magenta (LC15) with 228.34 μg/g dw, whereas the lowest was observed in Origami (LC3) with 79.62 μg/g dw. In different studies, the carotenoid composition in leaves of chrysanthemum (*Chrysanthemum morifolium*) revealed the presence of violaxanthin, neoxanthin, lutein, antheraxanthin, and β-carotene, from which lutein was found in the highest amount [83]. *Ornithogalum dubium*, a popular African ornamental plant, presented the highest lutein content (149.7 ± 35.4 μg/g), followed by lower values in β-carotene, violaxanthin, and neoxanthin [84]. A similar carotenoid profile was noticed in the leaves of *Petunia hybrida* cultivars, with the highest content in lutein, followed by β-carotene, (all-*E*)-violaxanthin, and (9′*Z*)-neoxanthin [85].

### 3.4. Qualitative and Quantitative Analysis of Individual Anthocyanins in Cyclamen Flowers

Anthocyanins are recognized for their antioxidant, anti-neurodegenerative, and antimutagenic properties, making them advantageous to be developed as key ingredients in the medicinal sector. They are found in flowers, leaves, fruits, foods, and, in several countries, are used as natural pigments [64]. These characteristics make them potential substitutes to artificial pigments in food, pharmaceutics, and cosmetics [29].

**Figure 4:**
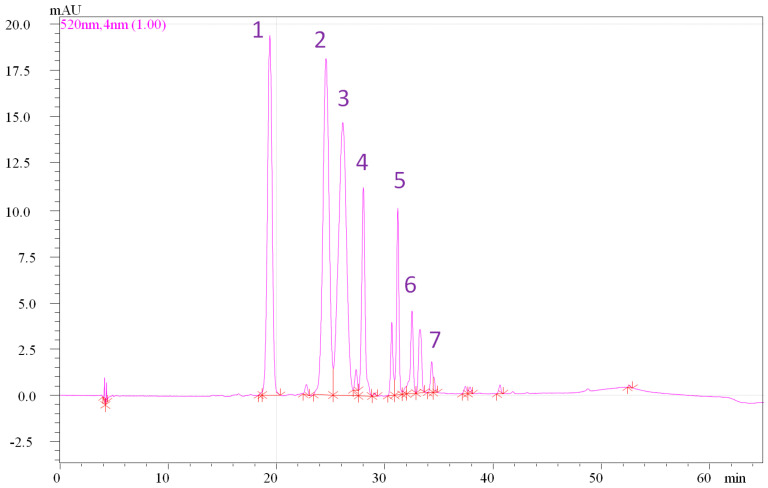
HPLC chromatogram of anthocyanins identified from *C. mirabile* flowers (FC6). Peak no. 1, Cyanidin 3,5-di-*O*-glucoside; 2, Peonidin 3,5-di-*O*-glucoside; 3, Malvidin 3,5-di-*O*-glucoside; 4, Peonidin 3-rutinoside; 5, Peonidin 3-*O*-glucoside; 6, Malvidin 3-*O*-glucoside; 7, Malvidin 3-rutinoside.

Based on the HPLC-PDA chromatograms and LC-MS data, the anthocyanins were identified and quantified (Supplementary Table S1; Figure 4). As shown in Table 3, the total anthocyanins content in the Cyclamen genotypes ranged from 203.0 µg/g fw (FC1) and 564.77 µg/g dw (FC6) to 411.6 µg/g fw (FC4) and 1673.60 µg/g dw (FC9). A total of seven anthocyanins were identified, including one cyanidin glycoside, three peonidin glycosides, and three maldivin glycosides. Among the identified anthocyanins, malvidin 3-*O*-glucoside and malvidin 3,5-di-*O*-glucoside were present at the highest levels in almost all genotypes. It was demonstrated that these anthocyanins inhibited nitric oxide production in the interferon-γ-activated/lipopolysaccharide raw 264.7 macrophage [86]. Meanwhile, peonidin and cyanidin 3,5-di-*O*-glucosides were present at the lowest values in FC2.1 and *C. mirabile* (FC6). In FC1, FC3, and *C. hederifolium* (FC18) high values of malvidin 3,5-di-*O*-glucoside were detected with protective effects against infection with *Helicobacter pylori*, cardiovascular disease, type 2 diabetes, metabolic disorders, and oral cancer [87]. Malvidin 3-*O*-glucoside was present at high levels in several *C. persicum* accessions, namely FC2, FC7, FC9, and FC15, respectively. Peonidin 3-*O*-glucoside and peonidin-rutinoside were detected at high levels in FC4. Peonidin 3-glucoside and cyanidin 3-glucoside were previously demonstrated to possess strong inhibitory effects on human breast carcinoma cell growth [88]. Finally, malvidin-rutinoside was detected in almost all genotypes except FC7, but only at lower levels. Previous studies reported the presence of high amounts of malvidin-3,5-diglucoside and lower amounts of cyanidin-3-neohesperidoside, peonidin-3-neohesperidoside, malvidin-3-rutinoside, and malvidin-3-glucoside in the flowers of *C. purpurascens* [33].

Conversely, in Slovenian *C. purpurascens* flowers, high amounts of malvidin 3,5-diglucoside and delphinidin-3-glucoside were detected [34]. These results are in accordance with Boase et al. (2010) which isolated the anthocyanins from the flowers of transgenic *C. persicum* through flavonoid 3′,5′-hydroxylase suppression [89]. Thus, in purple transgenic genotypes, high amounts of malvidin 3-5-di-*O*-diglucoside were noticed, whereas the red genotypes presented high levels of malvidin 3-*O*-glucoside. In the present study, the highest diversity and content in anthocyanins were noticed in the flowers of *C. persicum* Halios falbala (FC9) with 1673.60 µg/g dw, followed by Origami (FC3) with 1490.91 µg/g dw, whereas the lowest content was observed in *C. mirabile* (FC6), taking into account the high variety in peonidin and malvidin glycosides (564.77 µg/g dw). The anthocyanin profile of Cyclamen genotypes may provide a better understanding of the therapeutical value as the isolated compounds may potentially be used as health-promoting agents in the treatment of different ailments.

### 3.5. Total Phenolic Content (TPC), Total Flavonoid Content (TFC) and Antioxidant Assays

Polyphenols are plant secondary metabolites, recognized as natural antioxidants. On the basis of their chemical composition, they are classified as flavonoids and non-flavonoids [90]. Flavonoids can be classified into several sub-classes such as flavones, flavonols, flavanones, and isoflavonoids [91], whereas non-flavonoids generally comprise phenolic and ellagic acids, chalcones, anthraquinones, stilbenes, and ellagitannins [92].

According to Table 4, the highest values of TPC in the leaves of Cyclamen were found for LC6 (*C. mirabile*) (46.32 ± 0.14 mg/g GAE dw), followed by LC7 (38.80 ± 0.55 mg/g GAE dw). The lowest concentration was determined in LC2 (17.59 ± 0.49 mg/g GAE dw) and in *C. hederifolium* (10.03 ± 0.39 mg/g GAE dw), respectively. In the case of TFC, the highest level was noticed in LC9 (54.90 ± 0.27 mg/g QE dw), followed by *C. hederifolium* (47.59 ± 0.20 mg/g QE dw). The lowest levels were determined in LC6 (23.47 ± 0.28 mg/g QE dw) and LC15 (22.28 ± 0.16 mg/g QE dw), respectively. These results are in accordance with Turan and Mammadov (2018), who reported TPC values between 8.45 and8.95 mg/g GAE and TFC values between 47.25 and 92.63 mg/g QE in *C. alpinum* leaves [93]. The potential antioxidant activity of the leaves extract, analyzed through a DPPH assay, revealed the highest activity in LC1 (41.51 ± 0.10 mg/g Trolox dw), followed by LC2 (39.37 ± 0.02 mg/g Trolox dw), and the lowest in LC2.1 (22.78 ± 0.10 mg/g Trolox dw) and LC4 (13.86 ± 0.13 mg/g Trolox dw). Regarding the TEAC assay, the highest value was in *C. mirabile* (78.74 ± 0.50 mg/g Trolox dw) and the lowest in *C. hederifolium* (17.89 ± 0.71 mg/g Trolox dw), which might be due to the polyphenol content in the samples. Finally, the FRAP assay revealed significant differences between the extracts, with the highest value in LC1 (35.73 ± 0.21 mmol/g Fe^II^ dw) and the lowest in LC15 (17.02 ± 0.28 mmol/g Fe^II^ dw) which might be explained by the lower value in TFC. In accordance with the present results, Zengin et al. (2020) reported higher values of TFC (31.04 ± 0.26 mg/g RE) and lower values of TPC (22.27 ± 0.32 mg/g GAE) in the flowers of *C. cilicium* Boiss. and Heldr [94]. In a different study, Tian et al. (2018) showed that leaf berry extracts showed higher antioxidant activities compared with the fruit berry extracts as to the higher content of phenolic compounds [95].

In the case of flowers, the highest TPC values were found for FC15 (58.63 ± 0.17 mg/g GAE dw), followed by FC1 (50.68 ± 0.15 mg/g GAE dw). The lowest concentrations were noticed in *C. mirabile* (FC6, 30.81 ± 0.26 mg/g GAE dw) and FC7 (34.34 ± 0.20 mg/g GAE dw), respectively (Table 5). Regarding the TFC, high levels were obtained in FC2 (77.87 ± 0.25 mg/g QE dw) and FC9 (60.07 ± 0.33 mg/g QE dw), whereas low levels were registered in FC7 (34.84 ± 0.23 mg/g QE dw), followed by FC15 (34.45 ± 0.31 mg/g QE dw). The highest antioxidant potential measured by the DPPH assay was noticed in FC7 (40.35 ± 0.42 mg/g Trolox dw) and the lowest in FC15 (24.50 ± 0.08 mg/g Trolox dw). The TEAC assay revealed the highest value in FC1 (73.10 ± 0.45 mg/g Trolox dw), which might be explained by the relatively high polyphenol content, and the lowest in FC7 (43.51 ± 0.51 mg/g Trolox dw). Regarding the FRAP assay, the highest value was noticed in FC9 (38.30 ± 0.32 mmol/g Fe^II^ dw) and the lowest in FC4 (19.73 ± 0.11 mmol/g Fe^II^ dw) which might be due to the lower TPC and TFC values. In a different study, methanolic extracts of several *C. cilicium* parts were assessed to scavenge free radicals. Among the extracts, the roots showed the highest scavenging activity for DPPH and ABTS (94.28 ± 1.15 mg/g TE and 139.60 ± 0.11 mg/g TE, respectively), followed by the leaves (28.10 ± 0.54 mg/g TE and 31.27 ± 0.56 mg/g TE, respectively). A similar trend was also noticed for the reducing potential for CUPRAC and FRAP assays [94]. In a different study, the antioxidant activity of edible flowers before in vitro digestion was evaluated in comparison to their isolated bioactive compounds. Thus, the yellow variety Cosmos (*Cosmos sulphureus* Cav.) showed the highest phenolics content and ORAC activity correlated to the presence of major compounds (hesperidin and rutin). On the contrary, mini rose (*Rosa chinensis* Jacq.) exhibited the highest antioxidant activity in the DPPH and FRAP assays, correlated to the presence of pelargonidin 3,5-diglucoside and catechin [96]. Based on the present study results, the antioxidant properties vary greatly due to the differences in genotypes in terms of total phenolic and flavonoid content and the use of different scavenging activity methods which helps to better comprehend these actions.

### 3.6. Antimicrobial Activity—In Vitro Qualitative Study

Based on the phenolic and flavonoid contents, *C. persicum* accessions (LC9 and FC9), *C. mirabile* (LC6 and FC6), and *C. hederifolium* (LC18 and FC18) were selected for further antimicrobial potential against *S. aureus*, *Enter. fecalis*, *L. monocytogenes*, *B. cereus*, *E. coli*, *Salm. enteritidis*, *K. pneumonia*, and *C. albicans* strains, determined by the agar well diffusion method. Following the antimicrobial activity evaluation of the six compounds, it was observed that this was different depending on the bacterial strain and the product tested. The data obtained for the antimicrobial potential of the compounds are presented in Table 6.

An increased efficiency was observed for the five compounds (FC9) against Gram-negative bacteria. The potential of this extract against Gram-positive bacteria was moderate, with inhibition areas ranging from 7.29 to 8.64 mm. *B. cereus* and *C. albicans* were resistant for all the products evaluated. Due to the relatively low antibacterial activity, the MIC has not been further assessed (data not shown). These are in accordance with previous reports which demonstrated that Eastern sowbread (*C. coum* Boiss. and Heldr.) leaf aqueous extracts had no antibacterial effect against *P. aeruginosa*, *E. coli*, *S. aureus*, and *S. epidermidis* [97]. In different studies *C. coum* Miller subsp. coum extracts of flowers had an increased efficiency solely against *C. albicans* [98,99], highlighting the existing antibacterial differences even between similar genotypes. The potential antimicrobial activity of *C. persicum* Halios falbala (FC9) can be correlated with the total content of polyphenolic and flavonoid compounds that can act synergistically with the content of anthocyanins. The nature of the phenolic compounds present in the extract is very important and might potentiate the antibacterial action of the determined anthocyanins. Thus, FC9 has the highest total anthocyanin content and a high content of total flavonoids compared with the other samples (FC6 and FC18). The same is noticed regarding the efficiency of *C. mirabile* (FC6) against *Enter. fecalis* and *E. coli***,** which might be due to its diverse antocyanin composition, but also to its polyphenolic and flavonoid compounds. Previously, a strong correlation has been demonstrated between malvidin 3-*O*-glucoside and tested Gram-negative bacteria strains, with a significant effect against *E. coli*, *P. aeruginosa*, *P. vulgaris*, *S. enteritidis*, and *S. sonnei*, but not against *P. pneumonia* [100]. In a different study, malvidin 3-*O*-glucoside was found predominant in two Portuguese red grape varieties. The extracts demonstrated significant antibacterial activity against Gram-positive *E. faecium* and *B. cereus*, and Gram-negative *K. pneumonia*, with no effect against *S. aureus*, *E. faecalis*, *E. coli*, *P. aeruginosa*, and *S. entereditis* [101].

### 3.7. Cytotoxicity Assay

**Figure 5:**
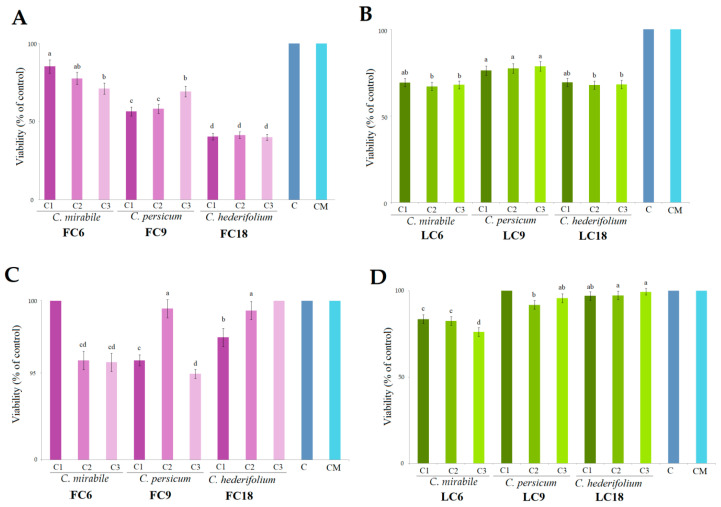
Inhibitory effects on MDA-MB-231 cell line of *Cyclamen* species (**A**) flowers and (**B**) leaves, and on BJ cell line of *Cyclamen* species (**C**) flowers and (**D**) leaves at three different concentrations (C1–C3) according to TPC (mg/g GAE) determined for each extracts. Different letters within a column indicate significant differences (*p* < 0.05). C—untreated cells, CM—Methanol.

As shown in Figure 5, the most intense in vitro cytotoxicity against MDA-MB-231 cells was noticed in *C. hederifolium* LC18 (56.71–69.35%) and FC18 (40.07–41.43%), respectively. A similar, but lower intensity was observed in the case of the *C. persicum* extracts LC6 (68.06 ± 1.06) and FC6 (77.42 ± 1.22), followed by *C. mirabile* LC9 (68.44 ± 0.81) and FC9 (78.09 ± 7.05), compared with the untreated control. The results could be associated to the presence of carotenoids in the *C. hederifolium* extract, which had significant levels of β-carotene, and in *C. mirabile* with increased levels of neoxanthin. On the contrary, the extracts tested against the BJ cell line were less effective in inhibiting proliferation compared to when they were against MDA-MB-231, thus demonstrating selective toxicity. Cyclamen flower extracts contain cyclamin, a saponin with reported anticancer properties, but with no significant protective anticlastogenic effects [10,102]. In the present study, the flowers of *C. hederifolium* (FC18) demonstrated the highest inhibitory activity against the MDA-MB-231 cell line with all used concentrations, which might be due to its high content in total phenolic and flavonoid content, but also due to its relatively high content in malvidin 3,5-di-*O*-glucoside (357.53 ± 3. 2 µg/g dw), malvidin 3-O glucoside (127.55 ± 0.7 µg/g dw), and malvidin rutinoside (95.07 ± 1.3 µg/g dw). In a different study, the main compound in *Malva sylvestris*, namely malvidin 3-glucoside, minimized the infection and inflammation processes of *Actinobacillus actinomycetemcomitans* in oral human cells [103]. Malvidin demonstrated inhibitory activities against the cell proliferation of stomach, colon, lung, breast, and CNS cancer cells, with values ranging from 40.5% to 75.7% [104]. Moreover, in the study conducted by Zengin et al. (2020), the methanolic *C. cilicium* extracts of flowers demonstrated strong cytotoxicity against the MCF-7 (3.17–5.01%) and MDA-MB-231 (2.79–5.29%) cell lines [94].

The leaves of *C. mirabile* (LC6) presented the highest values in neoxanthin (31.13 ± 2.2 dw) and violaxanthin (22.48 ± 0.87 µg/g dw), which may denote its cytotoxic activity in MDA-MB-231 and BJ cell lines. Additionally, the leaves of *C. hederifolium* (LC18) presented increased levels in lutein (135.10 ± 2.3 µg/g dw), β-carotene (55.40 ± 2.8 µg/g dw), and cis-β-carotene (10.46 ± 0.88 µg/g dw), accounting for their inhibitory effects against MDA-MB-231 and BJ cell lines. Consistent with the present results, it has been demonstrated that violaxanthin and neoxanthin were less effective for the reduction in cell viability of HeLa and A549 cells, whereas neoxanthin presented lower toxicity against normal MDCK cells compared with HeLa cells [105]. Recently, it was demonstrated that neoxanthin inhibits H_2_O_2_-induced cytotoxicity in HepG2 cells by increasing the anti-apoptotic protein expression, Bcl-2, and decreasing the Bax pro-apoptotic protein, and activating the protein expression of redox-sensitive transactivation factors [106]. Previous studies stated that lutein demonstrates antitumor effects against murine 4T1-induced breast cancer, and a notable decrease in the tumor weight and volume [107]. Furthermore, lutein derived from marigold (*Tagetes erecta* L.) strongly inhibited the proliferation of HeLa cells by 60% after 24 h and over 80% after 48 h of treatment, respectively [108]. Furthermore, among 15 tested dietary carotenoids, Kotake-Nara and his collaborators demonstrated that neoxanthin induced apoptosis in PC-3 human prostate cancer cells by reducing the expression of Bax and Bcl-2 proteins [109]. Therefore, Cyclamen flowers, particularly *C. persicum* and *C. hederifolium*, prove to be potential anticancer agents due to their significantly high content in carotenoids (i.e., lutein and β-carotene) and anthocyanins (i.e., malvidin 3-*O*-glucoside and cyanidin 3,5-di-*O*-glucoside).

### 3.8. Multivariate Statistics

#### 3.8.1. PCA and Correlations of the Phenolic Content with Bioactive Compounds and Antioxidant Activities

Multivariate analysis, such as the combination of cluster analysis and PCA, are techniques widely used for a precise classification of different extracts based on their chemical composition and bioactivities. PCA is the most commonly used method for performing multivariate analysis in flowers, leaves, and fruits of multiple plants [98,99], which is frequently compared with correlation coefficients [110].

**Figure 6:**
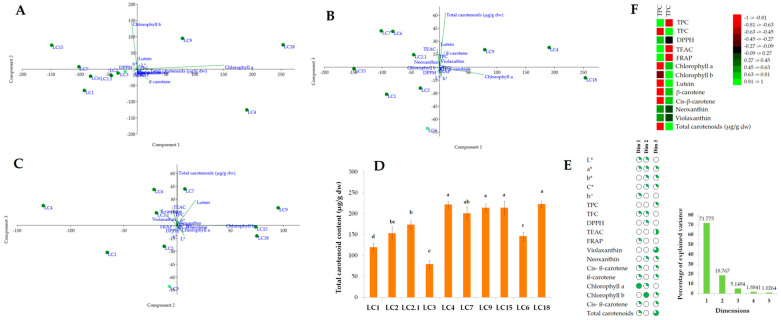
Exploratory multivariate analysis using PCA (**A**–**C**) with samples distribution into PC1 and PC2, PC1 and PC3, and PC2 and PC3, respectively; (**D**) Total carotenoids content of Cyclamen genotypes leaves; (**E**) Explained variance by each component and contribution of color measurements, antioxidant activities, and carotenoid profiles to the principal components; (**F**) Pearson’s correlation between total phenolic and flavonoid content, antioxidant activities, and carotenoid profiles.

Although statistically significant differences (*p* < 0.05) were noticed between the samples accounting to the chemical composition and bioactivities, multiple comparison analysis, using Tukey post hoc, displayed equal activities of several extracts. The dataset incorporating all Cyclamen extracts and their chemical composition, phenolic and flavonoid content, and antioxidant activities was subjected to PCA. On the basis of the PCA, a very good discrimination was observed (the first three principal components explained 95.67% of the data variance) and obtained based on the Cyclamen leaf extracts (Figure 6). PC1 and 2 explained 90.53% of the data variance and differentiated the samples based on their chlorophyll content and, to a lesser extent, based on their β-carotene and lutein content. Nonetheless, samples LC15 and *C. hederifolium* (LC18) were differentiated by their increased and similar chlorophyll content, and LC18 by its relatively high lutein content. PC1 and 3 explained 76.92% of the data variance and distinguished the samples based on the total carotenoids and TEAC activity. Samples LC6 and LC7 had similar lutein contents, from which LC6 possessed the highest neoxanthin content. Furthermore, LC1 and LC3 in the lower left quadrant had a similar carotenoid profile. PC2 and 3 accounted for 23.92% of the variance represented by the violaxanthin, β-carotene, and cis-β-carotene content in LC4 and the high content of lutein in LC9, antioxidant activities, and the lightness (L*) and shade (*H*°) of the *Cyclamen* leaves, particularly in the genotypes with silver patterns. Furthermore, according to the correlation coefficients (Figure 6F), the TPC strongly correlated (*p* < 0.001) to the chlorophyll and identified carotenoids, whereas TFC significantly correlated to the FRAP and TEAC assays. Correlation coefficients were also assessed to determine the relationship between the color parameters and carotenoids. Most individual compounds exhibited negative correlations with color measurements, and positive correlations were found between β-carotene and shade (*H*°) (data not shown).

**Figure 7:**
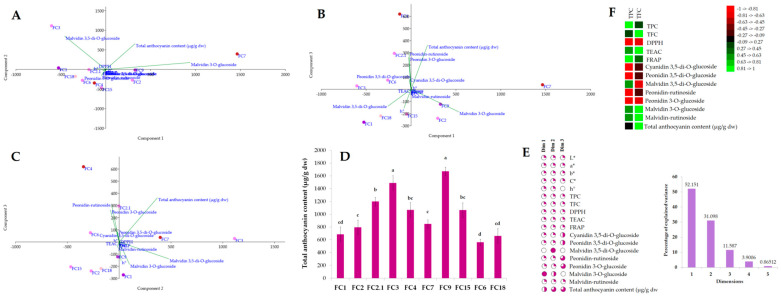
Exploratory multivariate analysis using PCA (**A**–**C**) with samples distribution into PC1 and PC2, PC1 and PC3, and PC2 and PC3, respectively; (**D**) Total anthocyanins content of Cyclamen genotypes flowers; (**E**) Explained variance by each components and contribution of color measurements, antioxidant activities, and anthocyanin profiles to the principal components; (**F**) Pearson’s correlation between total phenolic and flavonoid content, antioxidant activities, and anthocyanin profiles.

Regarding the flower extracts, the first three principal components explained 94.82% of the data variance (Figure 7). PC1 and 2 (83.24% of data variance) distinguished the flower samples based on their total anthocyanins, increased malvidin 3-*O*-glucoside, and peonidin rutinoside content, with similar colors and flowers shade (*H*°), particularly in FC4 and FC7. In addition, the negative b* coordinate values were highlighted owing to the violet-colored genotypes. PC1 and 3 accounted for 63.73% of the data variance, representing the samples with an increased peonidin-rutinoside and peonidin 3-*O*-glucoside content (FC4), similar a*, significant TFC, and antioxidant activities, particularly in FC1 and FC3. PC2 and 3 were 25.89% and 10.47%, highlighting the samples with distinct colors and peonidin 3,5 di-*O*-glucoside in samples FC2.1 and *C. mirabile* (FC6). According to the Pearson’s correlation matrix (Figure 7F) the TPC was strongly associated to the isolated anthocyanins, except malvidin glycosides, whereas the TFC significantly correlated to the DPPH assay, and malvidin 3,5-di-*O*-glucoside and peonidin 3-*O*-glucoside. The relationship between the color parameters and isolated compounds revealed positive correlations between L* and malvidin-rutinoside, and negative correlations between shade and peonidins (data not shown).

#### 3.8.2. HCA

Concomitantly, both HCA and PCA are generally used in studies involving bioactive compounds and functional activities. HCA is a broadly used method to assess the multivariate correlation between bioactive compounds and bioactivities of plants, foods, and other products [110,111]. Thus, to better discriminate the samples, an HCA was performed to differentiate the Cyclamen extracts based on isolated compounds and biological activities (*R* = 0.99).

**Figure 8:**
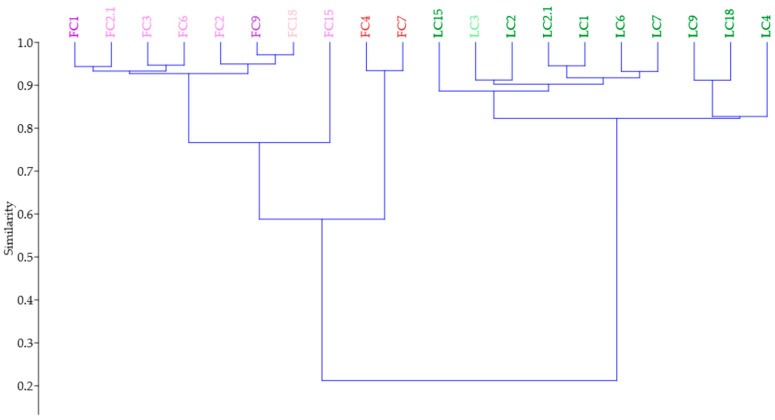
HCA of the Cyclamen genotypes based on isolated compounds and biological activities (Bray–Curtis similarity, *R* = 0.99).

The HCA organized the samples into two main clusters; in the first cluster, the ‘outliers’ FC3, FC4, and FC7 were separated from the others mostly due to their distinct anthocyanin profile, and the highest content was compared with the other samples (Figure 8). They possessed a similar TPC and TFC, and an increased peonidin rutinoside and peonidin 3-*O*-glucoside content in FC4, a high content of mavidin 3,5-di-*O*-glucoside in FC3, and an increased malvidin 3-*O*-glucoside content and DPPH activity in FC7. The following sub-cluster was comprised of the samples FC2, FC9, and FC15, from which FC2 had a high TFC content and FC15 an increased TFC content, whereas FC9 had an increased TFC and FRAP activity. The 2nd sub-cluster was comprised of *C. mirabile* (FC6) and FC2.1, which were differentiated from the other samples by their cyanidin 3,5-di-*O*-glucoside and peonidin 3,5-di-*O*-glucoside content and their similar DPPH and FRAP activities. The following FC1 and *C. hederifolium* (FC18) samples possessed similar anthocyanin profiles, from which FC18 had a similar TPC and TFC and increased FRAP activity. The second main cluster emphasized the leaf samples (LC4, LC9, and LC18) with increased chlorophyll a and b contents and a similar carotenoid profile and content. The following cluster separated LC15 due to its low antioxidant activity, whereas the other samples had similar TEAC activity. The last sub-cluster distinguished samples LC6 and LC7 by the similar DPPH activity. This shows that HCA clearly discriminated the Cyclamen extracts based on their chemical composition and activities.

#### 3.8.3. Dendrograms of HCA and Heatmap

**Figure 9:**
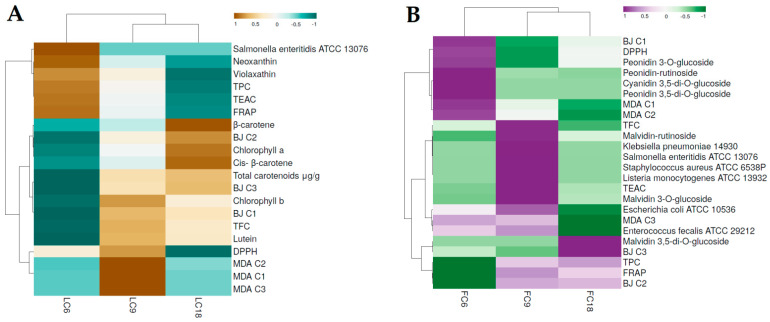
Hierarchical clustering and heatmap visualization of the *Cyclamen* leaves (**A**) and flowers (**B**) based on their biochemical composition, and antibacterial and anticancer activities.

HCA was performed to show the similarities and differences among flowers and leaves samples based on their antibacterial and citotoxicity activities. The heatmap and dendrograms of the HCA are shown in Figure 9. Following the importance score, LC6 was distinct due to its relatively low chlorophyll a and b content, but increased TPC, violaxanthin, and neoxanthin content. Furthermore, it exhibited antibacterial activity against *S. enteritidis* and lower inhibitory activity against BJ cells, with the highest effect in the 3rd concentration. Although in MDA cells it exhibited a higher cytotoxicity, no significant differences were noticed among the used concentrations. Samples LC9 and LC18 had no antibacterial activity and exhibited similar cytotoxicity at all concentrations against the tested cells (Figure 9A). Regarding the flowers (Figure 9B), FC6 was separated from the others by its antibacterial activity against *E. fecalis* and *E. coli* and its moderate cytotoxicity, with the best results using the 3rd concentration against MDA-MB-231 cells, whereas in BJ cells, no cytotoxicity was noticed using the 1st concentration and it was lower when using the others. Samples of FC9 exhibited the strongest antibacterial activity among the other extracts including the leaves, with the exception of *B. cereus* and *C. albicans*. It displayed the best cytotoxicity against MDA cells using the first two concentrations, whereas the 3rd concentration had the strongest effect against BJ cells. Lastly, FC18 only had relatively low antibacterial activity in *E. coli*, and had the highest cytotoxicity against MDA-MB-231 cells in all tested concentrations, whereas in BJ cells, a relatively low effect was noticed using the 1st concentration. These results suggest the use of Cyclamen flower extracts as antibacterial agents and also their application as future anticancer candidates. Further in vitro pharmacological investigations of specific isolated compounds are needed to better understand their use in several medicinal fields.

## 4. Conclusions

This study highlights the variations of the chemical compositions and biological activities between Cyclamen genotypes. It was found that the leaves of *C. persicum* accessions, *C. mirabile*, and *C. hederifoilium* are diverse in terms of their carotenoid profile and their antioxidant, antimicrobial, and antitumor activities. *Cyclamen* leaves possess significant carotenoids with particularly high contents of lutein and β-carotene content, with potential anticancer activities. The highest carotenoid content was noticed in *C. persicum* Merengue Magenta (LC15) with a 228.34 μg/g dw, whereas the lowest was observed in Origami (LC3) with a 79.62 μg/g dw. The highest content in anthocyanins was noticed in the flowers of *C. persicum* Halios falbala (FC9) with a 1673.60 µg/g dw, and the lowest in *C. mirabile* (FC6) with a 564.77 µg/g dw. The Cyclamen flowers exhibited increased antibacterial and antitumor activities, emphasizing their importance as a natural source of antioxidants. Thus, the highest antimicrobial activity was noticed in *C. persicum* (FC9) which might be due to its high content in malvidin 3-*O*-glucoside (663.88 ± 5.7 µg/g dw) and malvidin rutinoside (222.89 ± 2.6 µg/g dw). Additionally, the flowers of *C. hederifolium* (FC18) demonstrated the highest inhibitory activity against the MDA-MB-231 cell line with all used concentrations, which might be due to its high total phenolic and flavonoid content, but also due to its relatively high levels in malvidin 3,5-di-*O*-glucoside (357.53 ± 3.2 µg/g dw), malvidin 3-*O*-glucoside (127.55 ± 0.7 µg/g dw), and malvidin rutinoside (95.07 ± 1.3 µg/g dw). Overall, the compounds with the highest and broader antimicrobial activity proved to be malvidin glycosides, present in high amounts in FC9. Regarding the leaves, the highest cytotoxic activity was noticed in *C. mirabile* (LC6), which presented the highest values in neoxanthin (31.13 ± 2.2 dw) and violaxanthin (22.48 ± 0.87 µg/g dw) which may denote its cytotoxic activity against MDA-MB-231 and BJ cell lines.

The determination of the anthocyanins and carotenoids composition adds value in the study of the biologically active compounds found in the Cyclamen genotypes. Moreover, the relationships of the investigated chemical parameters and bioactivities were evaluated using multivariate statistics (PCA, HCA, and dendrograms), which could be a useful guidance for researchers and breeders in selecting genotypes based on their carotenoid and anthocyanin profile, but also on their potential therapeutic and ornamental usage. Further comprehensive evaluations of *Cyclamen* species are essential for progresses in their valorization, particularly via the characterization of secondary metabolites and evaluation of their bioactivities. These data may contribute to the increased knowledge of these traditional medicinal plants towards their efficient and safe utilization in the pharmaceutical and floricultural fields.

## Figures and Tables

**Table 1:** Colorimetric data of Cyclamen genotypes’ leaves and flowers.

Genotype	Sample ID	L*	a*	b*	C*	*H*°
**Leaves**						
***C. persicum*** **accessions**	LC1	50.97 ± 10.5 ^c^	−19.63 ± 4.4 ^c^	23.10 ± 4.4 ^c^	31.47 ± 2.0 ^c^	131.00 ± 10.9 ^c^
	LC2	51.37 ± 13.4 ^c^	−19.43 ± 4.6 ^c^	20.13 ± 4.2 ^c^	28.00 ± 6.2 ^c^	133.33 ± 2.0 ^c^
	LC2.1	57.97 ± 7.8 ^b^	−27.7 ± 2.9 ^d^	32.50 ± 2.5 ^b^	42.80 ± 3.5 ^b^	130.33 ± 2.0 ^c^
	LC3	73.23 ± 15.2 ^a^	−17.4 ± 3.6 ^c^	15.67 ± 1.9 ^d^	23.60 ± 1.1 ^d^	137.33 ± 5.4 ^b^
	LC4	48.93 ± 7.9 ^c^	−22.6 ± 3.3 ^cd^	11.43 ± 6.8 ^de^	26.47 ± 5.2 ^cd^	159.00 ± 14.0 ^a^
	LC7	37.83 ± 14.8 ^d^	−11.73 ± 2.2 ^b^	11.30 ± 2.5 ^e^	16.30 ± 3.3 ^e^	136.33 ± 1.4 ^b^
	LC9	64.7 ± 5.5 ^b^	−25.07 ± 2.0 ^d^	40.90 ± 10.4 ^a^	52.37 ± 6.0 ^a^	123.67 ± 9.4 ^d^
	LC15	61.23 ± 8.3 ^b^	−21.1 ± 7.3 ^cd^	16.33 ± 2.0 ^d^	27.30 ± 6.5 ^cd^	137.00 ± 10.2 ^bc^
***C. mirabile***	LC6	61.17 ± 5.4 ^b^	−5.4 ± 13.5 ^a^	20.47 ± 9.0 ^c^	29.63 ± 2.1 ^c^	91.00 ± 36.2 ^e^
***C. hederifolium***	LC18	76.67 ± 7.6 ^a^	−10.7 ± 1.7 ^b^	13.40 ± 6.6 ^de^	17.67 ± 6.1 ^e^	134.33 ± 9.33 ^c^
**Flowers**						
***C. persicum*** **accessions**	FC1	26.8 ± 3.6 ^c^	42.9 ± 6.2 ^c^	−8.6 ± 2.1 ^c^	43.9 ± 5.7 ^c^	348.0 ± 4.0 ^a^
	FC2	47.05 ± 16.1 ^ab^	31.2 ± 13.4 ^d^	−5.65 ± 5.4 ^c^	31.9 ± 14.1 ^d^	352.5 ± 6.5 ^a^
	FC2.1	41.75 ± 5.6 ^b^	55.6 ± 1.8 ^a^	−11.6 ± 2.0 ^d^	56.85 ± 2.1 ^a^	348.5 ± 1.5 ^b^
	FC3	48.3 ± 8.1 ^ab^	39.15 ± 3.5 ^c^	−16.85 ± 1.2 ^e^	42.6 ± 3.8 ^c^	336.5 ± 0.5 ^d^
	FC4	30.0 ± 2.4 ^bc^	47.45 ± 2.5 ^b^	22.05 ± 0.5 ^a^	52.3 ± 2.5 ^a^	25.0 ± 1.0 ^f^
	FC7	29.5 ± 9.8 ^bc^	48.2 ± 11.9 ^b^	27.15 ± 8.1 ^a^	55.35 ± 4.3 ^a^	29.0 ± 1.0 ^g^
	FC9	56.7 ± 18.9 ^a^	35.35 ± 14.5 ^d^	−6.6 ± 3.8 ^c^	36.0 ± 15.0 ^d^	350.0 ± 2.0 ^a^
	FC15	45.25 ± 13.6 ^ab^	49.3 ± 1.2 ^b^	0.15 ± 9.1 ^b^	50.1 ± 1.2 ^b^	170.0 ± 13.6 ^e^
***C. mirabile***	FC6	42.75 ± 7.3 ^ab^	32.5 ± 1.5 ^d^	−10.6 ± 2.4 ^d^	34.2 ± 2.2 ^d^	342.0 ± 3.0 ^c^
***C. hederifolium***	FC18	56.15 ± 13.1 ^a^	26.45 ± 19.2 ^e^	−5.95 ± 5.05 ^c^	27.15 ± 19.8 ^e^	349.5 ± 3.5 ^ab^

Values are represented as mean ± standard deviation. Within the same column, different letters indicate significant differences (*p* < 0.05); LC—Leaves of Cyclamen genotypes; FC—Flowers of Cyclamen genotypes.

**Table 2:** Carotenoids composition (μg/g fresh (fw) and dry weight (dw) ± SD) in Cyclamen genotypes leaves.

Species	Sample ID	Neoxantin μg/g		Violaxantin μg/g		Lutein μg/g		β-Carotene μg/g		Cis- β-Carotene μg/g		Total Carotenoids μg/g	
		fw	dw	fw	dw	fw	dw	fw	dw	fw	dw	fw	dw
***C. persicum*** **accessions**	LC1	6.66 ± 0.92 ^e^	9.66 ± 1.2 ^d^	6.23 ± 0.23 ^e^	9.66 ± 0.4 ^e^	48.07 ± 7.18 ^c^	69.78 ± 0.67 ^f^	17.57 ± 1.2 ^e^	25.50 ± 3.4 ^e^	3.84 ± 0.37 ^d^	5.57 ± 0.4 ^d^	82.4	119.58
	LC2	6.58 ± 0.34 ^ef^	10.41 ± 0.76 ^d^	6.09 ± 0.88 ^e^	9.63 ± 0.98 ^e^	56.59 ± 4.12 ^c^	89.57 ± 0.43 ^e^	23.08 ± 1.04 ^d^	36.53 ± 2.3 ^d^	4.73 ± 0.31 ^c^	7.48 ± 1.2 ^c^	97.09	153.65
	LC2.1	6.05 ± 0.31 ^f^	8.29 ± 3.4 ^de^	9.05 ± 0.45 ^c^	12.41 ± 0.56 ^d^	81.09 ± 9.43 ^ab^	111.21 ± 0.31 ^c^	25.78 ± 0.88 ^c^	35.35 ± 5.6 ^d^	5.20 ± 0.61 ^bc^	7.13 ± 1.6 ^c^	127.2	174.41
	LC3	5.17 ± 0.23 ^d^	7.27 ± 2.7 ^e^	3.93 ± 0.19 ^f^	5.53 ± 0.23 ^f^	31.91 ± 1.6 ^e^	44.92 ± 0.45 ^h^	12.67 ± 5.12 ^f^	17.83 ± 4.6 ^f^	2.87 ± 0.12 ^e^	4.04 ± 0.7 ^e^	56.58	79.62
	LC4	11.00 ± 0.89 ^b^	14.64 ± 5.1 ^c^	14.49 ± 0.23 ^a^	19.29 ± 1.1 ^b^	36.92 ± 1.5 ^d^	49.15 ± 0.43 ^g^	97.09 ± 0.23 ^a^	129.26 ± 12.6 ^a^	7.47 ± 0.09 ^a^	9.94 ± 2.2 ^a^	167.0	222.29
	LC7	6.78 ± 0.99 ^c^	10.13 ± 3.2 ^d^	10.24 ± 1.03 ^bc^	15.28 ± 2.3 ^c^	85.32 ± 3.78 ^ab^	127.38 ± 3.89 ^b^	30.43 ± 5.02 ^b^	45.43 ± 4.6 ^c^	6.06 ± 0.93 ^b^	9.04 ± 0.65 ^b^	138.83	195.84
	LC9	12.19 ± 0.72 ^b^	19.34 ± 1.8 ^b^	12.61 ± 1.45 ^b^	20.01 ± 2.87 ^b^	90.68 ± 9.05 ^a^	143.91 ± 0.78 ^a^	23.43 ± 0.32 ^d^	37.18 ± 6.7 ^d^	4.97 ± 0.17 ^bc^	7.88 ± 0.61 ^c^	143.9	207.28
	LC15	11.21 ± 1.26 ^b^	15.28 ± 0.8 ^c^	11.20 ± 1.14 ^b^	15.27 ± 2.3 ^c^	70.51 ± 9.04 ^b^	96.16 ± 1.2 ^d^	13.09 ± 0.97 ^f^	17.85 ± 3.12 ^f^	2.61 ± 0.21 ^f^	3.55 ± 1.1 ^e^	108.65	228.34
***C. mirabile***	LC6	21.49 ± 3.14 ^a^	31.13 ± 2.2 ^a^	15.52 ± 1.29 ^a^	22.48 ± 0.87 ^a^	74.97 ± 6.59 ^ab^	108.62 ± 1.89 ^c^	19.40 ± 4.14 ^e^	28.10 ± 3.7 ^e^	3.79 ± 0.72 ^d^	5.49 ± 1.5 ^d^	135.2	148.14
***C. hederifolium***	LC18	7.30 ± 0.85 ^c^	11.28 ± 1.9 ^d^	7.74 ± 0.61 ^d^	11.96 ± 0.35 ^d^	87.42 ± 9.23 ^a^	135.10 ± 2.3 ^b^	35.85 ± 5.45 ^b^	55.40 ± 2.8 ^b^	6.77 ± 0.84 ^b^	10.46 ± 0.88 ^a^	145.1	224.21

Values are represented as mean ± standard deviation. Within the same column, different letters indicate significant differences (*p* < 0.05); LC—*Cyclamen* leaves.

**Table 3:** Anthocyanin composition (μg/g fresh (fw) and dry weight (dw) ± SD) in Cyclamen genotypes flowers.

Species	Sample ID	Cyanidin 3,5-di-*O*-Glucoside		Peonidin 3,5-di-*O*-Glucoside		Malvidin 3,5-di-*O*-Glucoside		Peonidin-Rutinoside		Peonidin 3-*O*-Glucoside		Malvidin 3-*O*-Glucoside		Malvidin-Rutinoside		Total Anthocyanins (µg/g)	
		fw	dw	fw	dw	fw	dw	fw	dw	fw	dw	fw	dw	fw	dw	fw	dw
***C. persicum*** **accessions**	FC1	n.d.	n.d.	n.d.	n.d.	185.58 ± 2.42 ^b^	628.11 ± 3.1 ^b^	n.d.	n.d.	1.32 ± 0.1 ^f^	4.46 ± 0.3 ^h^	8.00 ± 0.3 ^f^	27.07 ± 0.5	8.10 ± 2.1 ^e^	27.41 ± 2.2 ^g^	203.0	687.07
	FC2	n.d.	n.d.	n.d.	n.d.	5.62 ± 1.31 ^g^	18.84 ± 1.2 ^h^	2.02 ± 0.3 ^e^	6.77 ± 0.1 ^e^	17.22 ± 0.6 ^d^	57.67 ± 0.8 ^e^	198.28 ± 4.1 ^b^	664.82 ± 3.9 ^b^	14.32 ± 1.5 ^d^	48.01 ± 1.8 ^e^	237.4	796.12
	FC2.1	68.14 ± 1.28 ^a^	255.52 ± 4.5 ^a^	73.06 ± 1.19 ^a^	273.97 ± 5.7 ^a^	62.17 ± 0.98 ^d^	233.13 ± 1.1 ^d^	32.11 ± 0.9 ^c^	120.41 ± 2.9 ^b^	47.08 ± 2.1 ^b^	176.55 ± 1.3 ^b^	24.03 ± 0.8 ^e^	90.11 ± 0.8 ^e^	13.14 ± 0.5 ^d^	49.27 ± 0.4 ^e^	319.7	1198.98
	FC3	n.d.	n.d.	n.d.	n.d.	286.46 ± 2.47 ^a^	1407.39 ± 11.7 ^a^	n.d.	n.d.	2.73 ± 0.1 ^e^	13.41 ± 0.2 ^g^	7.23 ± 0.2 ^f^	35.52 ± 1.4 ^h^	7.04 ± 0.3 ^e^	34.58 ± 0.3 ^f^	303.4	1490.91
	FC4	n.d.	n.d.	n.d.	n.d.	n.d.	n.d.	200.14 ± 3.7 ^a^	520.36 ± 4.4 ^a^	181.41 ± 2.7 ^a^	471.66 ± 4.7 ^a^	21.43 ± 0.1 ^e^	55.71 ± 1.1 ^g^	8.63 ± 1.9 ^e^	22.43 ± 1.1 ^h^	411.6	1070.18
	FC7	n.d.	n.d.	n.d.	n.d.	n.d.	n.d.	n.d.	n.d.	n.d.	n.d.	400.27 ± 6.6 ^a^	1673.6 ± 6.8 ^a^	n.d.	n.d.	400.2	852.234
	FC9	n.d.	n.d.	n.d.	n.d.	43.13 ± 2.1 ^e^	141.87 ± 1.9 ^e^	13.21 ± 0.3 ^d^	43.45 ± 2.1 ^c^	n.d.	n.d.	201.82 ± 3.5 ^b^	663.88 ± 5.7 ^b^	67.76 ± 3.4 ^a^	222.89 ± 2.6 ^a^	325.9	1673.60
	FC15	n.d.	n.d.	n.d.	n.d.	12.09 ± 0.8 ^f^	31.91 ± 1.1 ^g^	n.d.	n.d.	48.11 ± 2.4 ^b^	127.01 ± 2.5 ^c^	121.51 ± 6.1 ^c^	320.78 ± 2.8 ^c^	32.22 ± 2.4 ^b^	85.06 ± 2.2 ^c^	213.9	1072.10
***C. mirabile***	FC6	62.07 ± 0.89 ^b^	188.90 ± 3.1 ^b^	58.11 ± 1.15 ^b^	176.85 ± 5.1 ^b^	43.12 ± 2.11 ^e^	131.23 ± 3.8 ^f^	39.08 ± 1.5 ^b^	118.93 ± 3.6 ^b^	37.32 ± 1.5 ^c^	113.58 ± 2.2 ^d^	21.05 ± 2.3 ^e^	64.06 ± 2.6 ^f^	19.27 ± 2.3 ^c^	58.64 ± 1.7 ^d^	280.0	564.77
***C. hederifolium***	FC18	n.d.	n.d.	n.d.	n.d.	118.34 ± 4.1 ^c^	357.53 ± 3.2 ^c^	12.56 ± 0.3 ^d^	37.94 ± 1.2 ^d^	16.77 ± 2.1 ^d^	50.66 ± 0.7 ^f^	42.22 ± 2.7 ^d^	127.55 ± 0.7 ^d^	31.47 ± 3.1 ^b^	95.07 ± 1.3 ^b^	221.3	668.78

Values are represented as mean ± standard deviation. Within the same column, different letters indicate significant differences (*p* < 0.05); FC—*Cyclamen* flowers; n.d.—not detected.

**Table 4:** Total phenolic (TPC) and flavonoid (TFC) content, and antioxidant properties of tested *Cyclamen* leaves extracts.

Species	Sample ID	TPC (mg/g GAE dw)	TFC (mg/g QE dw)	DPPH (mg/g Trolox dw)	TEAC (mg/g Trolox dw)	FRAP (mmol/g Fe^II^ dw)
***C. persicum*** **accessions**	LC1	25.16 ± 0.67 ^de^	40.45 ± 0.10 ^d^	41.51 ± 0.10 ^a^	37.75 ± 0.45 ^e^	35.73 ± 0.21 ^a^
	LC2	17.59 ± 0.49 ^f^	25.26 ± 0.13 ^g^	39.37 ± 0.02 ^b^	35.89 ± 0.43 ^f^	34.74 ± 1.49 ^a^
	LC2.1	22.46 ± 0.44 ^e^	32.33 ± 0.15 ^f^	22.78 ± 0.10 ^g^	37.22 ± 0.15 ^e^	27.24 ± 0.76 ^c^
	LC3	26.72 ± 0.05 ^d^	35.49 ± 0.19 ^e^	27.14 ± 0.07 ^f^	40.80 ± 0.98 ^d^	21.09 ± 0.92 ^e^
	LC4	28.01 ± 0.32 ^c^	23.77 ± 0.21 ^h^	13.86 ± 0.13 ^h^	41.22 ± 0.75 ^d^	26.89 ± 0.92 ^c^
	LC7	38.80 ± 0.55 ^b^	45.17 ± 0.19 ^c^	28.98 ± 0.12 ^e^	61.60 ± 0.50 ^b^	19.82 ± 0.63 ^e^
	LC9	29.65 ± 0.32 ^c^	54.90 ± 0.27 ^a^	31.71 ± 0.12 ^d^	46.99 ± 0.35 ^c^	26.25 ± 0.64 ^c^
	LC15	23.19 ± 0.79 ^de^	22.28 ± 0.16 ^i^	38.61 ± 0.17 ^c^	30.65 ± 0.62 ^g^	17.02 ± 0.28 ^f^
***C. mirabile***	LC6	46.32 ± 0.14 ^a^	23.47 ± 0.28 ^h^	28.45 ± 0.16 ^e^	78.74 ± 0.50 ^a^	29.81 ± 0.21 ^b^
***C. hederifolium***	LC18	10.03 ± 0.39 ^g^	47.59 ± 0.20 ^b^	21.96 ± 0.16 ^g^	17.89 ± 0.71 ^h^	23.38 ± 0.58 ^d^

GAE—Gallic acid equivalents; QE—Quercetin equivalents; Values are represented as mean ± standard deviation. Within the same column, different letters within a column indicate significant differences (*p* < 0.05).

**Table 5:** Total phenolic (TPC) and flavonoid (TFC) content, and antioxidant properties of tested Cyclamen flower extracts.

Species	Sample ID	TPC (mg/g GAE dw)	TFC (mg/g QE dw)	DPPH (mg/g Trolox dw)	TEAC (mg/g Trolox dw)	FRAP (mmol/g Fe^II^ dw)
***C. persicum*** **accessions**	FC1	50.68 ± 0.15 ^b^	50.63 ± 0.35 ^d^	31.59 ± 0.08 ^d^	73.10 ± 0.45 ^a^	16.77 ± 0.12 ^h^
	FC2	47.23 ± 0.14 ^c^	77.87 ± 0.25 ^a^	27.23 ± 0.06 ^g^	69.80 ± 1.16 ^b^	30.19 ± 0.42 ^c^
	FC2.1	50.81 ± 0.31 ^b^	53.39 ± 0.23 ^c^	28.28 ± 0.12 ^e^	52.51 ± 0.54 ^f^	23.05 ± 0.80 ^e^
	FC3	42.38 ± 0.40 ^e^	43.67 ± 0.21 ^e^	39.17 ± 0.09 ^b^	52.22 ± 0.90 ^f^	21.30 ± 0.76 ^f^
	FC4	39.79 ± 0.20 ^f^	44.91 ± 0.26 ^e^	33.03 ± 0.09 ^c^	52.08 ± 0.14 ^f^	19.73 ± 0.11 ^g^
	FC7	34.34 ± 0.20 ^g^	34.84 ± 0.23 ^f^	40.35 ± 0.42 ^a^	43.51 ± 0.51 ^g^	23.07 ± 0.24 ^e^
	FC9	42.61 ± 0.16 ^e^	60.07 ± 0.33 ^b^	28.41 ± 0.08 ^ef^	58.99 ± 0.73 ^d^	38.30 ± 0.32 ^a^
	FC15	58.63 ± 0.17 ^a^	34.45 ± 0.31 ^f^	24.50 ± 0.08 ^h^	65.99 ± 0.81 ^c^	20.67 ± 0.21 ^f^
***C. mirabile***	FC6	30.81 ± 0.26 ^h^	52.20 ± 0.34 ^c^	28.84 ± 0.08 ^f^	53.70 ± 0.80 ^e^	24.84 ± 0.32 ^d^
***C. hederifolium***	FC18	44.33 ± 0.17 ^d^	49.41 ± 0.28 ^d^	28.61 ± 0.16 ^e^	54.13 ± 0.78 ^e^	35.88 ± 0.32 ^b^

Values are represented as mean ± standard deviation. Within the same column, different letters within a column indicate significant differences (*p* < 0.05).

**Table 6:** The in vitro qualitative screening—the disk diffusion test.

Used Strains	LC6	LC9	LC18	FC6	FC9	FC18	A	N	M
***St. aureus***	-	-	-	-	7.83	-	23.91	-	-
***Enter. fecalis***	-	-	-	7.26	8.64	-	26.76	-	-
***List. monocytogenes***	-	-	-	-	7.29	-	24.87	-	-
***B. cereus***	-	-	-	-	-	-	27.24	-	-
***E. coli***	-	-	-	10.27	12.31	7.25	-	25.87	-
***S. enteritidis***	7.27	-	-	-	10.24	-	-	27.42	-
***K. pneumoniae***	-	-	-	-	7.56	-	-	14.27	-
***C. albicans***	-	-	-	-	-	-	-	-	18.46

Note: A—amoxicillin, N—Norfloxacine, M—miconazole.

## Data Availability

Not applicable.

## Supplementary Materials

The following supporting information can be downloaded at: https://www.mdpi.com/article/10.3390/antiox11061126/s1; Table S1: Retention time (Rt), mass spectral data, and tentative identification of anthocyanins in Cyclamen flowers; Figure S1: Chemical structures of isolated carotenoids; Figure S2: Chemical structures of isolated anthocyanins.
