# Supplementary material for: Anthocyanins and Carotenoids Characterization in Flowers and Leaves of Cyclamen Genotypes Linked with Bioactivities Using Multivariate Analysis Techniques
Source: Antioxidants (Basel). 2022 Jun 7;11(6):1126. doi: 10.3390/antiox11061126 (PMC9220265; doi:10.3390/antiox11061126)

Supplementary Table S1. Retention time (Rt), mass spectral data, and tentative identification of anthocyanins in Cyclamen flowers

Peak no.	R_t (min)	UV λ_{max} (nm)	$[M+H]^+$ (m/z)	Compound	Chemical formula
1	19.39	282, 516	611	Cyanidin 3,5-di-O-glucoside	$C_{27}H_{31}O_{16}$
2	24.66	277, 330,516	625	Peonidin 3,5-di-O-glucoside	$C_{28}H_{33}O_{16}$
3	26.22	277, 343, 531	655	Malvidin 3,5-di-O-glucoside	$C_{29}H_{35}O_{17}$
4	28.12	282, 330, 521	609	Peonidin 3-rutinoside	$C_{28}H_{33}O_{15}$
5	31.33	282, 330, 516	463	Peonidin 3-O-glucoside	$C_{22}H_{23}O_{11}$
6	33.31	277, 348, 531	493	Malvidin 3-O-glucoside	$C_{23}H_{25}O_{12}$
7	34.45	277, 348, 531	639	Malvidin 3-rutinoside	$C_{29}H_{35}O_{16}$

Supplementary Figure S1. Chemical structures of isolated carotenoids

Neoxanthin

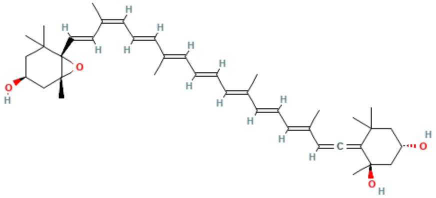

Violaxanthin

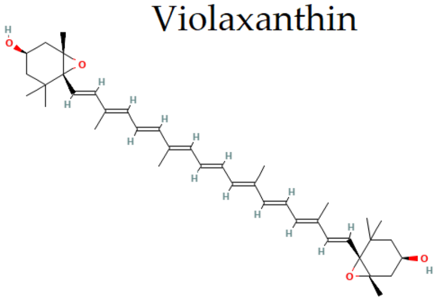

Lutein

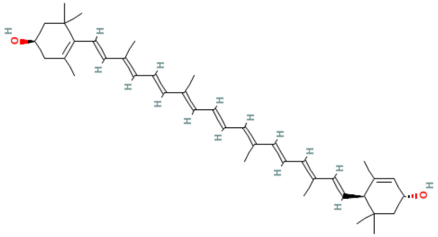

β-carotene

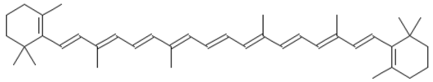

Cis-β-carotene

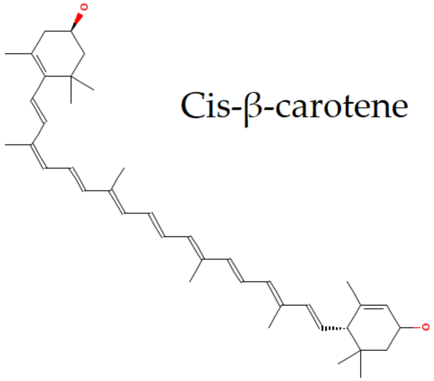

Supplementary Figure S2. Chemical structures of isolated anthocyanins

Cyanidin 3,5-di-O-glucoside

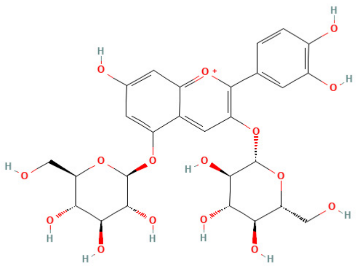

Malvidin 3,5-di-O-glucoside

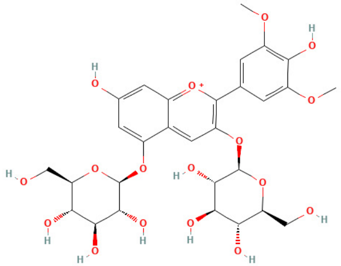

Peonidin 3-O-glucoside

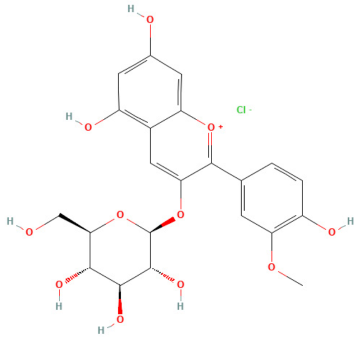

Malvidin 3-rutinoside

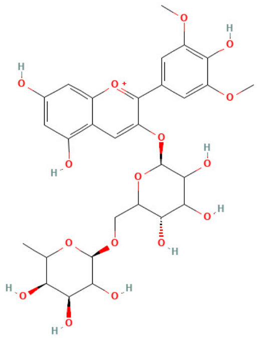

Peonidin 3,5-di-O-glucoside

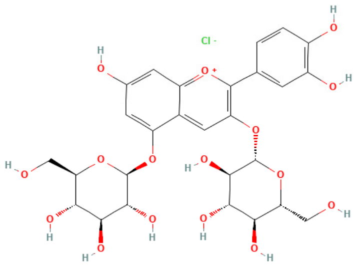

Peonidin 3-rutinoside

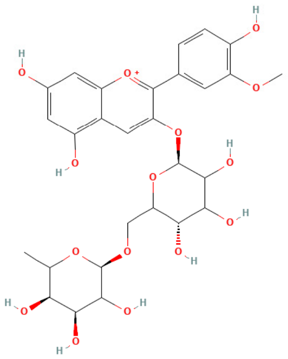

Malvidin 3-O-glucoside

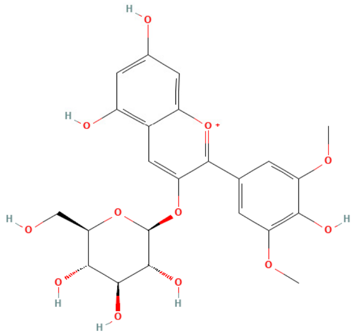

Supplement: Supplementary file 1 [file antioxidants-11-01126-s001.zip › antioxidants-1747268-supplementary.pdf]
